# Current Cigarette Smoking Is Associated With a High Seizure Frequency and Anxiety Symptoms in People With Epilepsy

**DOI:** 10.3389/fneur.2022.834694

**Published:** 2022-03-04

**Authors:** Rui Zhong, Zhuan Li, Xinyue Zhang, Qingling Chen, Weihong Lin

**Affiliations:** ^1^Department of Neurology, The First Hospital of Jilin University, Changchun, China; ^2^Department of Emergency, Linyi Central Hospital, Linyi, China; ^3^Department of Hepatology, Second People's Clinical College of Tianjin Medical University, Tianjin, China

**Keywords:** epilepsy, male, seizure frequency, cigarette smoking, anxiety symptoms

## Abstract

**Purpose:**

This study aims to answer the following questions: how many people with epilepsy (PWE) have cigarette smoking habits? Which demographic or clinical characteristics are associated with cigarette smoking? Is cigarette smoking related to depressive and anxiety symptoms in PWE?

**Methods:**

A total of 524 PWE were included in the final analysis. Demographic and clinical data were gathered and recorded. Smoking status was identified. The associations of smoking status with the clinical features of epilepsy and depressive and anxiety symptoms were evaluated by logistic regression models.

**Results:**

The overall prevalence of cigarette smoking was 23.5% (123 PWE) in this sample. In the multivariate logistic regression model, men (adjusted OR = 10.414, 95% CI: 5.552–19.535, *P* < 0.001), high seizure frequency (adjusted OR = 1.474, 95% CI: 1.043–2.084, *P* = 0.028), and anxiety symptoms (adjusted OR = 2.473, 95% CI: 1.483–4.112, *P* = 0.001) were shown to have independent associations with cigarette smoking in PWE.

**Conclusion:**

Our findings suggested that the overall prevalence of cigarette smoking was 23.5% in adults with epilepsy, which is slightly lower than that (26.6%) in general adults in China. In the present study, cigarette smoking was associated with men, high seizure frequency, and anxiety symptoms in PWE. Further prospective clinical studies with larger sample sizes are required to confirm our findings.

## Introduction

Epilepsy is a prevalent chronic brain disease that affects more than 70 million individuals worldwide (1). Epilepsy has been reported as an important cause of disability and mortality (2). Approximately 1,25,000 people with epilepsy die from their condition per year (3). Cigarette smoking is highly addictive, widely prevalent, and very hazardous (4). Researchers have established a strong association between cigarette smoking and an increased risk of heart disease, stroke, lung cancer, and oral cancer (5–8). Smoking is estimated to have killed 100 million people worldwide in the 20th century (4). Cigarette smoking may also be an important factor influencing the risk of seizures or epilepsy (9). It has been reported that cigarette smoking is common in adults with epilepsy. Picard et al. reported that the global prevalence of cigarette smoking was 32.1% among 429 people with epilepsy (PWE), significantly higher than the 19.0% smoking rate reported in the general population in Switzerland (10). However, a recent study in West China found that the prevalence of cigarette smoking among men with epilepsy was lower than that among general adult men in China, 25.5% compared to 52%, respectively (11).

The relationship between cigarette smoking and epilepsy is complex and remains controversial. In a prospective study of women aged 25 to 42 years in the Nurses' Health Study II, cigarette smoking was observed to be related to an increased risk of single or provoked seizures that were independent of stroke (9). The findings of a recent study also suggested that current smokers with epilepsy were almost four times more likely to have experienced a seizure in the previous year compared with their nonsmoking peers. Additionally, smokers with epilepsy were reported to have a higher risk of suffering from refractory epilepsy than nonsmoking PWE (12). However, in a recent study from West China, a beneficial effect of cigarette smoking on seizure control was found (11).

The relationship between cigarette smoking and depressive and anxiety symptoms is also not yet fully understood in PWE. Epilepsy has been related to an increased risk of psychiatric comorbidities, including depression and anxiety (13, 14). Smoking has also been identified as an important risk factor for major depression (15, 16). It is necessary to investigate the association among cigarette smoking, clinical features of epilepsy, and depressive and anxiety symptoms. Thus, this study aimed to answer the following questions: how many people with epilepsy have cigarette smoking habits? Which demographic or clinical characteristics are associated with cigarette smoking? Is cigarette smoking related to depressive and anxiety symptoms in people with epilepsy?

## Methods

### Participants

A crosssectional study among people with epilepsy in Northeast China was carried out. Individuals with epilepsy who were treated and followed at the Epilepsy Outpatient Clinic of The Department of Neurology at The First Hospital of Jilin University were consecutively enrolled in this study between April 2017 and January 2020. Individuals were invited to participate in the current study if they met the following inclusion criteria: (1) 18 years of age or older, (2) definite diagnosis of epilepsy according to the International League Against Epilepsy (ILAE) diagnostic criteria by a neurologist (17), (3) sufficient physical, mental, and language ability to complete the interview and questionnaire, and (4) willingness to participate and provide written informed consent. Exclusion criteria were: (1) a history of nonepileptic seizures (assessed *via* medical record review), (2) a severe brain disease other than epilepsy (e.g., dementia and Parkinson's disease) or a serious psychiatric disease (e.g., lifetime depression or schizophrenia), or (3) a history of intellectual disability or language disability.

The information was obtained *via* medical records and patient self-reports during a face-to-face interview. A total of 581 PWE were initially invited to participate in the study, and 57 PWE were excluded from the analytic sample: 21 PWE declined to participate, 9 PWE were not able to complete the questionnaire, 11 PWE lacked the required data, and 16 PWE had major comorbid diseases. A total of 524 participants were included in the final analysis. This study was performed in accordance with the Helsinki Declaration and was approved by the Ethics Committee of the First Hospital of Jilin University (approval no: 2017-326). The participants provided a signed written informed consent.

### Data Collection

The demographic and clinical data were gathered and recorded from a face-to-face interview with a standardized questionnaire supplemented with medical records. We recorded the following demographic data: age, sex, marital status, educational level, and occupation. The clinical features of the epilepsy comprised age at first seizure onset, epilepsy duration, epilepsy type, seizure frequency over the last year, and antiseizure medication (ASM) polytherapy. The epilepsy type was classified as generalized or partial seizures. Seizure frequency over the last year was divided into seizure-free (0/month), < 1/month, and ≥ 1/month. The number of ASMs ≥ 2 was defined as ASM polytherapy. Depressive and anxiety symptoms were assessed by two trained clinicians (Rui Zhong and Xinyue Zhang) using the Neurological Disorders Depression Inventory for Epilepsy (C-NDDI-E; Chinese version) (18) and the 7-item Generalized Anxiety Disorder-7 questionnaire (GAD-7; Chinese version) (19).

### Smoking Status

We gathered information on smoking status as never smokers, former smokers, and current smokers (9). We considered as a “current smoker” any subject who had smoked at least one cigarette per day (or any other way of using tobacco at least once a day) for at least the past 6 months and “former smoker” as any subject who had smoked at least one cigarette per day for at least 6 months but was no longer smoking. Nonsmokers were never smokers and formal smokers.

### Assessment of Depressive and Anxiety Symptoms

The NDDI-E scale is a rapid and user-friendly clinical instrument to screen for depressive symptoms in PWE (18). The Chinese version of the NDDI-E has been validated by Tong et al. (20). It consists of six self-rated questions, with scores ranging from 1 to 4. The total score is obtained by summing the scores for the six questions, and a higher C-NDDI-E score indicates more serious depressive symptoms. The PWE were classified as having depressive symptoms by means of a standard cutoff point >12 (20). The GAD-7 scale is a valid and efficient tool for the screening of anxiety disorders and assessing their severity in clinical practice (19). In this study, the Chinese version of the GAD-7 was used to screen for anxiety symptoms in PWE (21). This questionnaire has seven self-rating questions, with scores ranging from zero to three. A higher GAD-7 score indicates more serious anxiety symptoms. A cutoff score of >6 indicated anxiety symptoms in PWE (21).

### Statistical Analyses

Continuous variables are presented as the means ± standard deviation (SD), and categorical variables are presented as frequencies and percentages. In the comparison of the two independent groups, Student's *t*-test or the Mann–Whitney U test were used for continuous variables, depending on the data distribution, which was assessed using the Kolmogorov–Smirnov test, and Chi-square tests were used for categorical variables. The Spearman correlation analysis was performed to evaluate the correlation among smoking status, clinical features of epilepsy, and depressive and anxiety symptoms. The associations of cigarette smoking with the clinical features of epilepsy and depressive and anxiety symptoms were evaluated by univariate and multivariate logistic regression models. A difference of *P* < 0.05 was deemed statistically significant, and all statistical tests were two-sided. Data were analyzed with IBM SPSS v. 26.0.

## Results

A total of 524 people with epilepsy were included, with a mean age of 38.41 years. The percentage of men was 58.6%. The overall prevalence of cigarette smoking was 23.5% (123 individuals) in this sample. Sixty-two (11.8%) PWE were former smokers and 339 (64.7%) were never smokers. Their demographic information, clinical features of epilepsy, and depressive and anxiety symptoms are described in Table 1.

**Table 1:** Patient characteristics.

**Variables**	
Age, mean ± SD	38.41 ± 16.46
Male, *n* (%)	307 (58.6)
Married, *n* (%)	330 (63.0)
Unemployed, *n* (%)	160 (30.5)
**Educational level, *n* (%)**
University and above	146 (27.9)
Middle school	315 (60.1)
Primary school and below	63 (12.0)
Age at first seizure onset, mean ± SD	33.89 ± 18.24
Disease duration, mean ± SD	4.76 ± 7.49
**Epilepsy type, *n* (%)**
Focal	436 (83.2)
Generalized	88 (16.8)
**Seizure frequency over the last year, *n* (%)**
Seizure-free	89 (17.0)
<1/month	282 (53.8)
≥1/month	153 (29.2)
ASM polytherapy, *n* (%)	49 (9.4)
Depressive symptoms, *n* (%)	97 (18.5)
Anxiety symptoms, *n* (%)	133 (25.4)
**Smoking status, *n* (%)**
Current smoker	123 (23.5)
Non-smokers	401 (76.5)
Fomer smokers	62 (11.8)
Never smoked	339 (64.7)

In this cohort, current smokers were more likely to be men than nonsmokers (*p* < 0.001). Educational level showed significant differences between the group of current smokers and nonsmokers (*p* = 0.001). Additionally, current smokers tended to experience a higher seizure frequency over the last year than nonsmokers (*p* = 0.014). Current smokers were also more likely to suffer from anxiety symptoms than nonsmokers (*p* = 0.011). There were no significant differences in age, marriage status, occupational status, age at first seizure onset, epilepsy duration, epilepsy type, seizure frequency, ASM polytherapy, or depressive symptoms between current smokers and nonsmokers (*p* > 0.05). For details see Table 2.

**Table 2:** Comparison of the demographic and clinical characteristics of current smokers and nonsmokers with epilepsy.

**Variables**	**Non-smokers (*n* = 401)**	**Current smokers (*n* = 123)**	***p*-value**
Age, mean ±SD	37.92 ± 16.85	40.02 ± 15.06	0.217
Male, *n* (%)	197 (49.1)	110 (89.4)	<0.001
Married, *n* (%)	248 (61.8)	82 (66.7)	0.333
Unemployed, *n* (%)	127 (31.7)	33 (26.8)	0.308
**Educational level**, ***n*** **(%)**			
University and above	111 (27.7)	35 (28.5)	0.01
Middle school	251 (62.6)	64 (52.0)	
Primary school and below	39 (9.7)	24 (19.5)	
Age at first seizure onset, mean ± SD	33.31 ± 18.86	35.76 ± 15.97	0.059
Disease duration, mean ± SD	4.86 ± 7.58	4.44 ± 7.22	0.321
**Epilepsy type**, ***n*** **(%)**			
Focal	328 (81.8)	108 (87.8)	0.119
Generalized	73 (18.2)	15 (12.2)	
**Seizure frequency over the last year**, ***n*** **(%)**			
Seizure-free	78 (19.5)	11 (8.9)	0.014
<1/month	214 (53.4)	68 (55.3)	
≥1/month	109 (27.2)	44 (35.8)	
ASM polytherapy, *n* (%)	38 (9.5)	11 (8.9)	0.859
Depressive symptoms, *n* (%)	68 (17.0)	29 (23.6)	0.098
Anxiety symptoms, *n* (%)	91 (22.7)	42 (34.1)	0.011

Spearman correlation analysis among smoking status, demographics, and clinical features of epilepsy, and depressive and anxiety symptoms of PWE are shown in Table 3. Male sex (*r* = 0.347 and *p* < 0.001), high seizure frequency (*r* = 0.119 and *p* = 0.007) and anxiety symptoms (*r* = 0.112 and *p* = 0.011) were positively correlated with cigarette smoking by PWE. Age at first seizure onset was slightly positively correlated with smoking, but after correction did not reach statistical significance (*r* = 0.083 and *p* = 0.059). No correlation was found between smoking status and the other variables (*p* > 0.05).

**Table 3:** Correlation between smoking status and other variables among PWE.

**Variables**	**Smoking status**	
	***r***	***p*-value**
Age	0.078	0.075
Male	0.347	<0.001
Married	0.042	0.334
Unemployed	0.045	0.309
Educational level	−0.054	0.219
Age at first seizure onset	0.083	0.059
Disease duration	−0.043	0.322
Epilepsy type	−0.068	0.119
Seizure frequency	0.119	0.007
ASM polytherapy	0.008	0.859
Depressive symptoms	0.072	0.099
Anxiety symptoms	0.112	0.011

In the univariate logistic regression model, men (OR = 8.762, 95% CI: 4.774–16.082, *P* < 0.001), high seizure frequency (OR = 1.55, 95% CI: 1.135–2.118, *P* = 0.006), and anxiety symptoms (OR = 1.766, 95% CI: 1.138–2.742, *P* = 0.011) were significantly associated with cigarette smoking by PWE. Further analyses with a multivariate logistic regression model were performed to identify the independent factors associated with cigarette smoking by PWE. Men (adjusted OR = 10.414, 95% CI: 5.552–19.535, *P* < 0.001), high seizure frequency (adjusted OR = 1.474, 95% CI: 1.043–2.084, *P* = 0.028), and anxiety symptoms (adjusted OR = 2.473, 95% CI: 1.483–4.112, *P* = 0.001) still had independent associations with cigarette smoking in PWE. The odds of men with epilepsy having cigarette smoking habits were 10.414 times greater than those of women with epilepsy. The odds of PWE with high seizure frequency having cigarette smoking habits were 1.474 times greater than those of PWE with low seizure frequency. The odds of PWE with anxiety symptoms having cigarette smoking habits were 2.473 times greater than those of PWE without anxiety symptoms. For details, see Table 4.

**Table 4:** Univariate and multivariate logistic regression analyses for independent factors associated with cigarette smoking in PWE.

**Variables**	**Unadjusted OR (95% CI)**	***p*-value**	**Adjusted OR (95% CI)**	***p*-value**
Age	1.008 (0.996–1.02)	0.217	–	–
Male	8.762 (4.774–16.082)	<0.001	10.414 (5.552–19.535)	<0.001
Married	1.234 (0.806–1.888)	0.333	–	–
Unemployed	1.264 (0.805–1.984)	0.308	–	–
Educational level	0.786 (0.564–1.094)	0.153	–	–
Age at first seizure onset	1.007 (0.996–1.018)	0.194	–	–
Disease duration	0.992 (0.964–1.021)	0.587	–	–
Epilepsy type	0.624 (0.344–1.133)	0.121	–	–
Seizure frequency	1.55 (1.135–2.118)	0.006	1.474 (1.043–2.084)	0.028
ASM polytherapy	1.066 (0.527–2.154)	0.859	–	–
Depressive symptoms	1.511 (0.924–2.469)	0.1	–	–
Anxiety symptoms	1.766 (1.138–2.742)	0.011	2.473 (1.483–4.122)	0.001

## Discussion

The present study revealed that the overall prevalence of current cigarette smoking was 23.5% in adults with epilepsy, which is slightly lower than that (26.6%) in general adults in China (11). Men and high seizure frequency were found to be related to cigarette smoking in adults with epilepsy. Additionally, in this cohort, current smokers were more likely to suffer from anxiety symptoms than nonsmokers.

China has a high prevalence of cigarette smoking, and the general smoking rate among Chinese men has reached 52.1% (11). In Chinese culture, cigarette smoking is a commonly accepted social activity and has many social benefits (22). Smoking could facilitate social connectedness and integration among Chinese people (23). The smoking rate of adults with epilepsy has been previously reported to vary among different populations (10–12). Compared with the previously reported cigarette smoking rates, the 23.5% of PWE with cigarette smoking in this sample is similar to the 25.5% one reported by Gao et al. (11). However, the prevalence of cigarette smoking (23.5%) in our study was lower than the 46.74% reported by Johnson et al. (12) and the 32.1% reported by Torriani et al. (10). Additionally, Torriani et al. observed that the prevalence of smoking among PWE was 32.1%, which was significantly higher than that (19.0%) in the general population in Switzerland (10). However, this result was not supported by our data. We found in this cohort, that the smoking rate was 23.5% in adults with epilepsy in Northeast China, which is lower than that (26.6%) in general Chinese adults.

Different from some developed countries, the Chinese patient–doctor mode is quite paternal, which implies that PWE are more likely to follow their doctor's instructions in their daily life, and doctors always advise PWE to quit smoking after the diagnosis. Additionally, a recent study in West China reported similar findings (11). The reasons for the conflicting results on the smoking rate of PWE are uncertain. This may be due to the differences in methodology of assessing smoking status, inclusion criteria, and sample sizes among these studies. Another possible explanation could be the differences in social and environmental factors across countries. For example, policies prohibiting smoking in public places and increasing cigarette prices *via* tax increases have been identified to influence people's smoking behavior (24, 25). These social and environmental strategies appear to differ across countries.

This current study supported a relationship between cigarette smoking and men with epilepsy. The odds of men with epilepsy having cigarette smoking habits were 10.414 times greater than those of women with epilepsy. Existing literature suggests that smoking rates among men are much higher in Chinese cities, indicating that men in China smoke far more than women, which is in line with our findings (26). The sex differences in the smoking status may be influenced by both individual and socioecological factors (27, 28). Additionally, the univariate analysis suggested that a lower educational level was a risk factor for cigarette smoking. However, this association was not significant in multivariate logistic regression analysis. Data from the Catalan Health Survey have indicated that men with a higher educational level tend to have a lower probability of being a smoker than those with an education of less than primary school; however, a reverse trend is present in women (29).

Our data suggested that PWE suffering from a higher seizure frequency were more likely to be current smokers, indicating a potential relationship between cigarette smoking and the risk of seizures. The odds of PWE with a high seizure frequency having cigarette smoking habits were 1.474 times greater than those of PWE with a low seizure frequency. There is a possible biological explanation that nicotine, as an excitatory neurotransmitter, could lead to an increased release of glutamate. High doses of nicotine can induce convulsions in predisposed mice (30). Additionally, cigarette smoking is related to poor sleep quality and sleep disturbance, which may have an indirect impact on the risk of seizures (31–33).

The impact of cigarette smoking on the risk of having a seizure or epilepsy remains an important question. There are multiple studies on the associations of cigarette smoking with seizure/epilepsy risk in PWE, but the results are controversial and even conflicting (9, 11, 12). In a prospective cohort of women aged between 25 and 42 years, Dworetzky et al. reported that current smokers tended to have an increased risk of seizures compared to nonsmokers after adjustment for stroke and other potential confounding factors (9). Past smoking was not associated with the risk of seizures but was associated with a modestly increased risk of epilepsy (9). Similarly, Johnson and his colleagues found that current cigarette smoking was related to an almost 4-fold increase in the risk of experiencing a seizure over the past year compared to nonsmoking in a cohort of people with various types of epilepsy (12). However, a beneficial effect of cigarette smoking on seizure control was established by Gao et al. (11). The various findings might be partly explained by the different inclusion criteria and study samples across studies. For example, Zhou et al. explored the role of cigarette smoking in seizure control in men with epilepsy (11). However, over 80% of the patients had refractory epilepsy in the study by Johnson et al. (12). Further prospective clinical studies with larger sample sizes are needed to clarify the exact role of smoking in seizure risk and seizure control in PWE.

Mental illness may be related to smoking habits. In our study, the odds of PWE with anxiety symptoms having cigarette smoking habits were 2.473 times greater than those of PWE without anxiety symptoms. A recent systematic review also found that baseline depression or anxiety was associated with some type of later smoking behavior in nearly half of the included studies (34). The self-medication hypothesis suggests that people turn to smoking to alleviate their depressive and anxiety symptoms (35, 36). It is known that psychiatric comorbidities, including depression and anxiety, are common in people with epilepsy (13, 37). To our knowledge, this is the first study to explore the relationship between cigarette smoking and depressive and anxiety symptoms in PWE. Interestingly, cigarette smoking was associated with an increased prevalence of anxiety symptoms. A recent systematic review provided similar evidence that baseline depression/anxiety increased the risk of some type of later smoking behavior in the general population (34). A possible explanation is that the high prevalence of anxiety in patients related to the personal and social burden due to epilepsy may lead to an increased risk of smoking dependence.

Several limitations should be noted in the current study. First, we employed a crosssectional study design; thus, definitive conclusions about causality cannot be made. Additional prospective investigation is warranted to confirm how smoking and epilepsy impact one another over time. In addition, prospective studies are needed to investigate the effect of smoking cessation on the risk of seizures in PWE. Second, depressive and anxiety symptoms were diagnosed according to the scores of the C-NDDI-E and GAD-7 scales in this study. Clinical diagnosis is the gold standard. The C-NDDI-E is not a substitute for clinical interviews and the Diagnostic and Statistical Manual (4th ed.) diagnosis, but it is a reliable, validated, and widely used self-report measure of depressive symptoms in mainland China (13). Third, the possibility of residual confounding factors cannot be excluded, although we adjusted for factors that may be related to smoking status among PWE. Additionally, the smoking status and other data of the participants were mainly based on self-report and are prone to responder bias. Finally, we did not evaluate other tobacco consumption methods, such as the Chinese hookah or bong.

## Conclusion

In conclusion, our findings suggested that the overall prevalence of cigarette smoking was 23.5% in adults with epilepsy, which is slightly lower than that (26.6%) in general adults in China. In the present study, cigarette smoking was associated with men, high seizure frequency, and anxiety symptoms among PWE. Additional prospective clinical studies with larger sample sizes are required to confirm our findings.

## Data Availability Statement

The raw data supporting the conclusions of this article will be made available by the authors, without undue reservation.

## Ethics Statement

The studies involving human participants were reviewed and approved by the Ethics Committee of the First Hospital of Jilin University. The patients/participants provided their written informed consent to participate in this study.

## Author Contributions

RZ and WL conceived and designed the study. RZ and XZ were involved in data acquisition. RZ, QC, and ZL analyzed the data and wrote the manuscript. All authors contributed to the article and approved the submitted version.

## Funding

This work was supported by a grant from the Programme of Jilin University First Hospital Clinical Cultivation Fund (LCPYJJ2017006).

## Conflict of Interest

The authors declare that the research was conducted in the absence of any commercial or financial relationships that could be construed as a potential conflict of interest.

## Publisher's Note

All claims expressed in this article are solely those of the authors and do not necessarily represent those of their affiliated organizations, or those of the publisher, the editors and the reviewers. Any product that may be evaluated in this article, or claim that may be made by its manufacturer, is not guaranteed or endorsed by the publisher.
